# Identification of *FLRT2* as a key prognostic gene through a comprehensive analysis of TMB and IRGPs in BLCA patients

**DOI:** 10.3389/fonc.2023.1229227

**Published:** 2024-02-29

**Authors:** Yaling Tao, Xiaoling Yu, Huaiwei Cong, Jinpeng Li, Junqi Zhu, Huaxin Ding, Qian Chen, Ting Cai

**Affiliations:** ^1^ Research Institute, Ningbo No.2 Hospital, Ningbo, China; ^2^ Ningbo Institute of Life and Health Industry, University of Chinese Academy of Sciences, Ningbo, China; ^3^ Department of Research and Development, Thorgene Co., Ltd., Beijing, China; ^4^ Department of Pathology, Ningbo Clinical Pathology Diagnosis Center, Ningbo, China; ^5^ Research Institute, Ningbo Hangzhou Bay Hospital, Ningbo, China

**Keywords:** bladder cancer, prognosis, TMB, IRGPs, FLRT2, methylation

## Abstract

**Introduction:**

The tumor immune environment and immune-related genes are instrumental in the development, progression, and prognosis of bladder cancer (BLCA). This study sought to pinpoint key immune-related genes influencing BLCA prognosis and decipher their mechanisms of action.

**Methods and results:**

We analyzed differentially expressed genes (DEGs) between high- and low- tumor mutational burden (TMB) groups. Subsequently, we constructed a reliable prognostic model based on immune-related gene pairs (IRGPs) and analyzed DEGs between high- and low-risk groups. A total of 22 shared DEGs were identified across differential TMB and IRGPs-derived risk groups in BLCA patients. Through univariate Cox and multivariate Cox analyses, we highlighted five genes - *FLRT2, NTRK2, CYTL1, ZNF683, PRSS41* - significantly correlated with BLCA patient prognosis. Notably, the *FLRT2* gene emerged as an independent prognostic factor for BLCA, impacting patient prognosis via modulation of macrophage infiltration in immune microenvironment. Further investigation spotlighted methylation sites - cg25120290, cg02305242, and cg01832662 - as key regulators of *FLRT2* expression.

**Discussion:**

These findings identified pivotal prognostic genes in BLCA and illuminated the intricate mechanisms dictating patient prognosis. This study not only presents a novel prognostic marker but also carves out potential avenues for immunotherapy and targeted therapeutic strategies in BLCA. By demystifying the profound impact of immune-related genes and the tumor immune environment, this study augments the comprehension and prognostic management of bladder cancer.

## 1 Introduction

Bladder cancer (BLCA), a globally acknowledged prevalent malignancy (1), had an estimated incidence of 81,180 and was responsible for 17,100 deaths in the United States in 2022 (2, 3). The majority of patients (i.e., 70%–75%) are diagnosed as having non-muscle-invasive bladder cancer (NMIBC) at onset, while approximately 20%–25% of patients are diagnosed as having muscle-invasive bladder cancer (MIBC) (4). The patients with NMIBC often experience high recurrence (50%–70%) and progression (10%–30%) rates (5). Advanced and metastatic BLCA, an invariably fatal disease, exhibits 5-year overall survival and progression-free survival rates of less than 15% (6, 7). Despite advancements in BLCA treatment through immunotherapy, targeted therapies, and neoadjuvant chemoimmunotherapy, high mortality and recurrence rates persist (8, 9). Hence, the critical need for new, efficient prognosis targets.

In recent years, gene expression profiling techniques, including gene microarrays and RNA sequencing, have become widely used in the search for biomarkers associated with BLCA prognosis (10–12). However, a major limitation of this approach is its inability to account for correlations between genes. Interestingly, the tumor mutational burden (TMB), which reflects the total load of neoantigens, displays a robust correlation with immunotherapy responsiveness (13–15). Additionally, immune-related genes, pivotal in modulating the immune system, have been deemed crucial in the development and progression of cancer (16, 17). Contemporary research is increasingly focusing on immune-related gene pairs (IRGPs) studies to identify prognostic biomarkers for patients (18, 19).

In this study, we conducted a combination analysis of TMB and IRGPs to identify prognostic genes in BLCA. We examined differentially expressed genes (DEGs) in high- and low-TMB groups, constructed a risk model using IRGPs, and then performed an analysis of the DEGs between the high- and low-risk groups. The common DEGs between the different TMB and risk groups were subsequently isolated. Through this process, we identified a key gene, fibronectin leucine-rich transmembrane protein 2 (*FLRT2*) and clarified its prognostic significance in BLCA.

*FLRT2*, a member of the FLRT family of proteins, contains 10 leucine-rich repeat (LRR) domains and a transmembrane domain (20). Flintoff KA et al. discovered that *FLRT2* interacted with fibronectin through either repulsion or adhesion, behaving as an adhesion molecule, suggesting a potential connection between *FLRT2* and cancer metastasis (21). Recent studies have shown that *FLRT2* expression correlates negatively with the long-term survival of colorectal cancer patients and that *FLRT2* facilitates the aggressiveness of colorectal cancer (22). However, the role of *FLRT2* in BLCA remains unexplored. In this study, we clarified the identification process of *FLRT2* and the impact of this gene on BLCA prognosis, thereby augmenting the understanding of its role in disease progression and its potential as a therapeutic target in BLCA.

## 2 Materials and methods

### 2.1 Sample data collection and processing

Publicly available data were utilized for this comprehensive analysis. The data of transcriptome cohorts and clinical features were obtained from The Cancer Genome Atlas (TCGA-BLCA, *n* = 433, https://portal.gdc.cancer.gov/) and the Gene Expression Omnibus (GSE31684, *n* = 93, https://www.ncbi.nlm.nih.gov/geo/). A list of 1,776 immune genes and their functional classification was retrieved from ImmPort (https://www.immport.org/shared/home), which was accessed on 25 November 2020. Ensembl ID conversion and extraction of the relevant clinical data were performed using Strawberry Perl (5.30.11). Other data processing was conducted using R (version 3.6.1; The R Foundation for Statistical Computing, Vienna, Austria).

### 2.2 Simple nucleotide variation data analysis and visualization

The BLCA simple nucleotide variation (SNV) data from TCGA, which is referred to as the “masked somatic mutation” subtype, were processed using VarScan software. The R package “maftools” [16] was employed to analyze and visualize the somatic variants in mutation annotation format (MAF). The germline DNA variants were removed, and the remaining mutation cases were used to determine the TMB using the R package “maftools”.

### 2.3 Copy number variation data processing

The BLCA copy number variation (CNV) data, referred to as the “masked copy number segment” type, were downloaded from the TCGA database. The data processing was conducted with Strawberry Perl (5.30.11), and visualization was performed using the R package “RCircos”.

### 2.4 Construction of a prognostic IRGP risk model based on the TCGA cohort

For sample-specific pairwise comparisons, two immune-related genes were paired, and if the first immune-related gene exhibited higher levels of expression than the second, the two genes were combined into one immune-related gene pair (IRGP) and assigned a score of 1; otherwise, the score was set to 0. Utilizing the initial candidate IRGPs, the prognostic model was constructed by univariate and multivariate Cox proportional risk regression. Finally, 62 gene pairs were used to define the final model. The optimal cutoff value for the IRGP index, which was analyzed by receiver operating characteristic (ROC) curves for 5-year overall survival (OS), enabled the division of patients into high-risk and low-risk groups. Kaplan–Meier (K–M) survival curves were employed to compare the differences in OS between the high- and low-risk groups, and log-rank tests were applied.

### 2.5 Acquisition of differentially expressed genes

The DEG analysis was conducted with R package “limma” and visualized with R package “pheatmap” in this study. The gene filtering condition was set to a false-discovery rate (FDR) < 0.05.

### 2.6 Functional enrichment analysis

Gene ontology (GO) function and Kyoto Encyclopedia of Genes and Genomes (KEGG) pathway enrichment were performed using the R packages “clusterProfiler”, “org.Hs.eg.db”, and “enrichplot”. Visualization was achieved with R package “ggplot2”. The gene set enrichment analysis (GSEA) was carried out by gsea-3.0.jar and Strawberry Perl (5.30.11). Significant enrichment criteria were set as a *p*-value < 0.05 and a FDR < 0.05.

### 2.7 Infiltration of 22 types of immune cells in BLCA

To calculate the infiltration level of 22 types of immune cells, cell type identification by estimating relative subsets of RNA transcripts (CIBERSORT) was used to evaluate and predict the enrichment of the immune cells. The R packages “CIBERSORT” and “Leukocyte signature matrix” were used to analyze the percentage of 22 immune cells’ infiltration in each sample. The *p*-values less than 0.05 were considered significant.

### 2.8 TIMER and GEPIA database analysis

The expression of the key gene *FLRT2* and overall survival were analyzed using GEPIA (Gene Expression Profiling Interactive Analysis; http://gepia2.cancer-pku.cn/). The relationship between the CNV level of *FLRT2* and immune cell infiltration was evaluated using TIMER (Tumor IMmune Estimation Resource; https://cistrome.shinyapps.io/timer/).

### 2.9 RNA isolation and real-time polymerase chain reaction

Tumor samples of six BLCA patients were obtained from Ningbo Clinical Pathology Diagnosis Center, Ningbo, China. The BLCA patients were divided into long-survival and short-survival patient groups by varying survival durations (long survival: overall survival > 5 years; short survival: overall survival < 2 years). The total RNA of tumor samples was extracted using RNeasy FFPE Kit (QIAGEN, 73504) according to the standard protocol. RNA concentration was measured by a NanoDrop™ 2000 Spectrophotometer (Thermo Fisher Scientific, Waltham MA, USA) by calculating from the optical density at 260 nm (OD_260_). Then the RNA was reverse transcribed to cDNA with the PrimeScript™ RT Reagent Kit (Perfect Real Time) (Takara RR037A) following the manufacturer’s instructions. Then RT-PCR was performed with a SLAN-96S real-time PCR thermal cycler, using a SYBR™ Green mixture (Takara RR820A) for relative mRNA quantification. Glyceraldehyde 3-phosphate dehydrogenase (GAPDH) was used as the loading control. Each qPCR reaction was conducted in triplicate. The following primers were used: GAPDH forward—5′-GATTCCACCCATGGCAAATTC-3′; GAPDH reverse—5′-CTGGAAGATGGTGATGGGATT-3′; *FLRT2* forward—5′-TGTGCCTGTTGGGCTTCCT-3′; and *FLRT2* reverse—5′-CGGCGATACCCTTGTTGGT-3′. The thermal cycling was conducted with the following parameters: 10 s at 60°C, 10 s at 95°C, 10 s at 95°C and 45 s at 58°C for 40 cycles, and 2 min at 60°C. The 2−ΔΔCt method was used to estimate the relative mRNA expression of the target genes.

### 2.10 Immunohistochemistry

The antibody against *FLRT2* was purchased from Invitrogen (anti-*FLRT2* antibody, PA5–32122). Immunohistochemistry (IHC) of six BLCA tumor samples was performed according to the manufacturer’s instructions. Four 5-μm sections were cut from each case. After dewaxing, slides were boiled with 1 mM EDTA pH 8.0 followed by 15 min at a sub-boiling temperature. The slides were washed with phosphate-buffered saline three times for 5 min each. The slides were subsequently quenched in 3% hydrogen peroxide for 15 min, and then blocked with 10% goat serum for 10 min. The slides were incubated overnight at 4°C with the primary antibody diluent (1: 2,000). The slides were then incubated with a biotinylated secondary antibody, per the manufacturer’s recommendation, for 30 min. Antibody binding was visualized with 3,3′-diaminobenzidine (DAB; ZLI-9018, OriGene).

### 2.11 Gene methylation and sites methylation correlation statistics with gene expression

The BLCA gene methylation data were downloaded from the TCGA database. Gene methylation was statistically performed using the R package “limma”, and site methylation was analyzed using Strawberry Perl. For the gene methylation difference analysis, the Wilcoxon test was used for data validation. Heatmaps and correlation charts were generated in R.

### 2.12 Statistical analysis

All statistical analyses and graphics were performed using R software (version 3.6.1). The data analysis was conducted using a Student’s *t*-test or the Wilcoxon rank-sum test. Least absolute shrinkage and selection operator (lasso) Cox regression analysis with 10-fold cross-validation was carried out using the R package “glmnet”. The statistical significance was set as a *p*-value < 0.05.

## 3 Results

### 3.1 The workflow design of the current study

**Figure 1:**
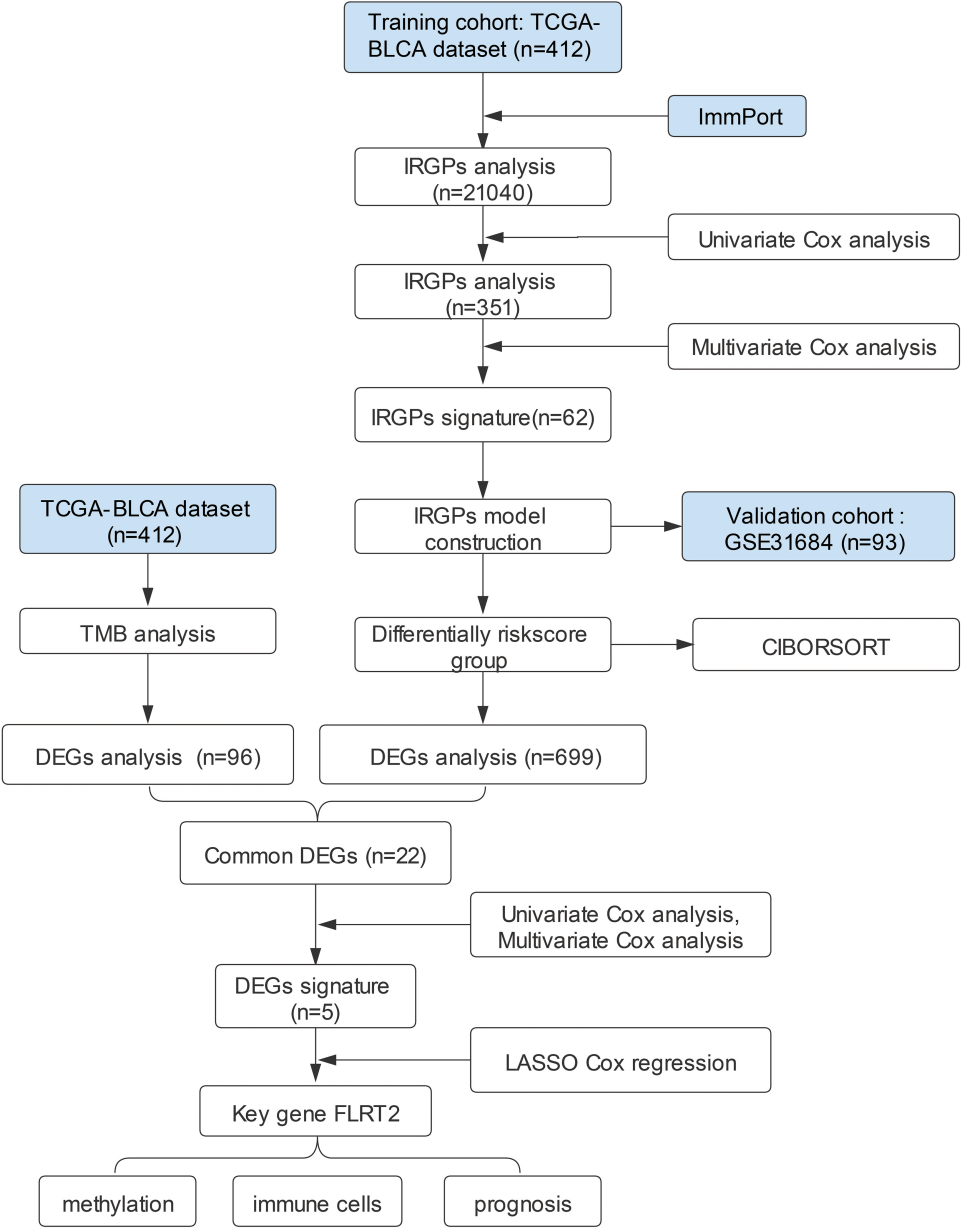
The workflow design of the current study. TCGA, the Cancer Genome Atlas; BLCA, bladder urothelial carcinoma; GEO, Gene Expression Omnibus; IRGPs, immune-related gene pairs; DEGs, differentially expressed genes; TMB, tumor mutational burden; CIBORSORT, cell-type identification by estimating relative subsets of RNA transcripts; LASSO, least absolute shrinkage and selection operator.

The workflow design of this study is presented in **Figure 1**. A total of 412 BLCA patients were included in this study for analysis. We obtained SNV data from the TCGA database, calculated tumor mutational burden (TMB) values, and further divided the patients into high- and low-TMB groups. The transcriptome sequencing data from the TCGA database and a list of immune-related genes from the ImmPort website were utilized to analyze BLCA IRGPs. A total of 62 IRGPs were identified through univariate and multivariate Cox analysis, which allowed the construction of a prognostic model for BLCA patients (**Table S1**). Based on the risk score calculated by this model, patients were divided into high- and low-risk groups. The common DEGs between the high- and low-risk groups and the high- and low-TMB groups that affected the prognosis of BLCA patients were analyzed. Finally, *FLRT2*, a new key gene affecting BLCA prognosis, was identified, and its mechanism of action on patient prognosis was further investigated.

### 3.2 The landscape of somatic mutations in BLCA patients

**Figure 2:**
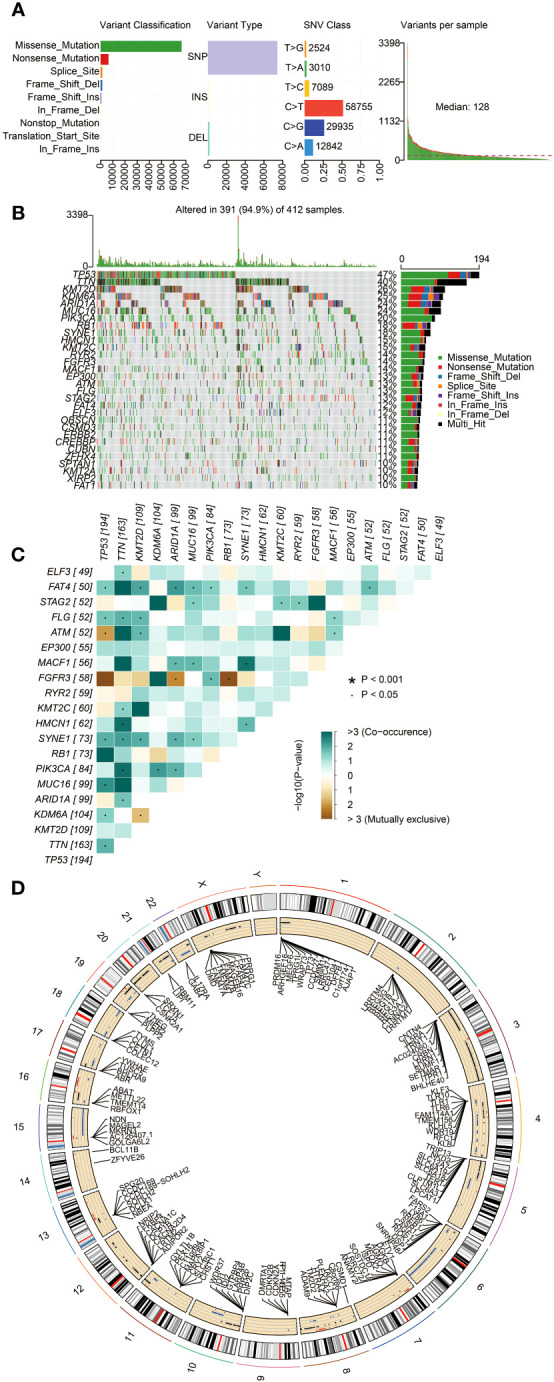
The landscape of somatic mutations in BLCA patients. **(A)** The diagram in order (from left to right): frequency of variant classification; frequency of variant types; frequency of SNV classes; and the level of gene variants in each sample. **(B)** Waterfall plot displaying the top 30 mutated genes in BLCA. The left panel shows different mutation types in each sample of BLCA. The right panel presents the genes ordered by their mutation frequencies. **(C)** The co-occurrence and mutually exclusive correlation with mutated genes. The solid black bullet (•) denotes a *p*-value < 0.05. The asterisk (*) denotes a *p*-value < 0.001. **(D)** Circos plot showing genes with copy number variation (CNV). The outer circle indicates the differential chromosomes, and the inner circle displays the genes with CNV. The red dot denotes increased CNV. The blue dot denotes decreased CNV.

The somatic mutation data of BLCA were downloaded from the TCGA database. The variant classification of somatic mutations included missense mutations, nonsense mutations, splice sites, frameshift deletions, frameshift insertions, in-frame deletions, non-stop mutations, translation initiation sites, and in-frame insertions. The proportion of missense mutations was the highest. The number of single nucleotide polymorphisms (SNPs) was more than deletions (DELs) and insertions (INSs). The SNV type with C > T was the most common (**Figure 2A**). The frequency of variants for each sample was also calculated and displayed. The detailed mutation information for the 30 genes with the highest mutation frequency for all the sample (*n* = 412) is shown in **Figure 2B**. The mutation frequency of *TP53*, *TTN*, *KMT2D*, *KDM6A*, *ARID1A*, *MUC16*, and *PIK3CA* was above 20%. The co-occurrence and mutually exclusive associations across the top 20 mutated genes are exhibited in **Figure 2C**. The CNVs of BLCA patients were mainly located on chromosomes 4, 6, and 15 (**Figure 2D**). These results indicated the presence of a somatic mutation profile in BLCA, which may affect the prognosis of BLCA patients by influencing the tumor immune microenvironment and the sensitivity of tumor therapy.

### 3.3 The role of TMB in prognosis of BLCA patients

**Figure 3:**
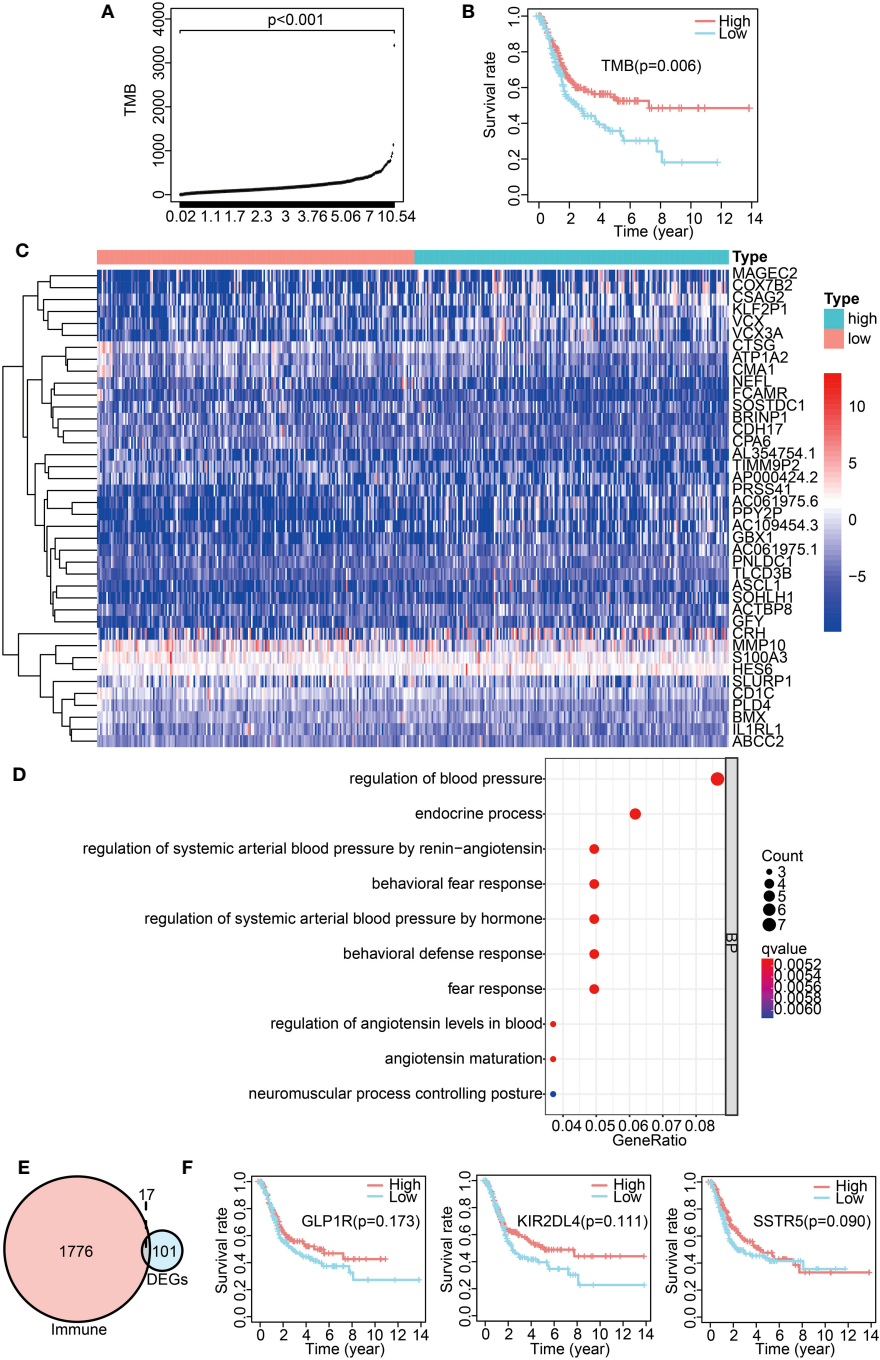
Analysis of TMB-derived DEGs and their correlation with BLCA prognosis. **(A)** TMB value in each sample. **(B)** Survival analysis between high- and low-TMB groups by Kaplan–Meier survival curves. **(C)** Heatmap exhibiting the top 40 DEGs between the high- and low-TMB groups. **(D)** GO enrichment analysis of DEGs. **(E)** Venn diagram of immune-related DEGs between the high- and low-TMB groups. **(F)** Kaplan–Meier curves displaying an association of *GLP1R*, *KIR2DL4*, and *SSTR5* expression with overall survival.

To explore the impact of TMB on the prognosis of BLCA patients, we calculated the TMB value for each sample, and then categorized the samples into high- and low-TMB groups based on the median TMB value. The TMB values for each sample are presented in **Figure 3A**. The figure shows statistically significant differences between the high- and low-TMB groups (*p* < 0.001). The Kaplan–Meier analysis indicated a significant correlation between TMB and prognosis (*p* = 0.006), revealing that BLCA patients in the high-TMB group had a more favorable prognosis than those in the low-TMB group (**Figure 3B**). To investigate whether or not TMB affects BLCA prognosis by modulating the immune microenvironment, we analyzed differences in infiltrated immune cells between the high- and low-TMB groups. The results indicated significant differences in the proportions of memory B cells, CD8 T cells, resting memory CD4 T cells, activated memory CD4 T cells, regulatory T cells (Tregs), M1 macrophages, resting mast cells, and neutrophils between the two groups (**Figures S1A**, **S1B**). To find the key immune genes that affect prognosis, we first identified 101 DEGs between the high- and low-TMB groups (**Figure S2A**). The heatmap displaying the top 40 DEGs is presented in **Figure 3C**. The gene ontology (GO) enrichment analysis showed that these DEGs were primarily related to the regulation of blood pressure (**Figure 3D**). On comparing the 101 DEGs with the ImmPort database, we identified 17 of these DEGs as being immune-related genes (**Figure 3E**). To assess their impact on patient prognosis, we performed univariate Cox analysis for these 17 immune DEGs. As a result, *GLP1R*, *KIR2DL4*, and *SSTR5* were found to be significantly associated with the prognosis of BLCA patients (**Table S2**). However, the subsequent multivariate Cox analysis did not show a statistically significance between the expression of these three genes and overall survival (OS) (**Figure 3F**). These findings suggest that these TMB-derived immune-related DEGs have a role in shaping the immune landscape; however, their effects on the prognosis of BLCA patients might be multifaceted.

### 3.4 Construction of a prognostic risk model with IRGPs for BLCA patients

While the association analysis between TMB-derived immune genes and BLCA prognosis failed to identify a clear target, we shifted our focus on constructing a prognostic risk model based on IRGPs. For this, TCGA transcriptome data were designated as a training cohort, while the GEO transcriptome data were used for validation. From the ImmPort database, we extracted 1,713 immune-related genes. The IRGPs were constructed from these genes. To ensure the robustness of the risk model, only IRGPs with a median absolute deviation (MAD) more than 0.5 were retained. This filtration resulted in a comprehensive list of 21,040 IRGPs. With subsequent univariate and multivariate Cox regression analysis on the IRGPs within the TCGA cohort, 62 IRGPs were retained and used for constructing a prognostic risk model. A majority of these IRGPs were associated with pathways such as BCR signaling, cytokine receptors, antimicrobials, and cytokine-related molecules (**Table S1**).

**Figure 4:**
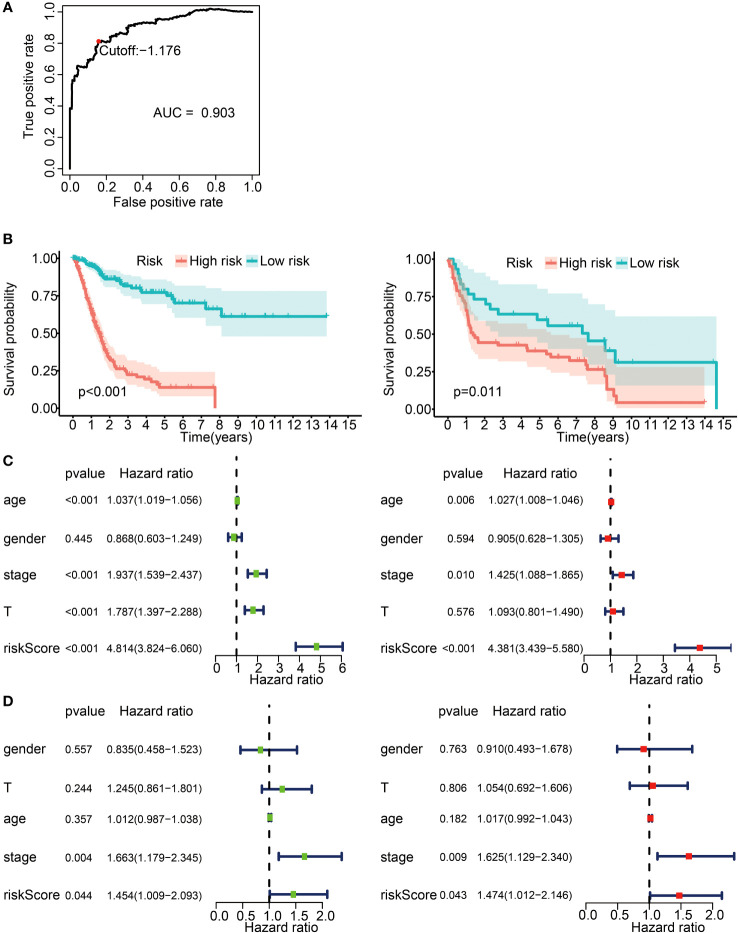
Prognostic risk model construction with 62 IRGPs for BLCA patients. **(A)** ROC curve for the IRGP risk model in the TCGA cohort. A risk score of −1.176 was used as a threshold for the IRGPs risk model to classify patients into high- and low-risk groups. ROC, receiver operating characteristic; AUC, area under curve. **(B)** Survival analysis of patients in the high- and low-risk groups with Kaplan–Meier survival curves. Left panel: the TCGA cohort. Right panel: the GSE31684 cohort. **(C)** Forest plot for univariate and multivariate Cox analysis in the TCGA cohort. Stage, clinical staging; T, T status of TNM staging; riskScore, risk score calculated from the prognostic risk model. Left: univariate Cox analysis. Right: multivariate Cox analysis. **(D)** Forest plot for univariate and multivariate Cox analysis in the GSE31684 cohort. Left panel: univariate Cox analysis. Right panel: multivariate Cox analysis.

We then classified the patients into high- and low-risk groups based on the optimal risk model threshold (−1.176). Impressively, the AUC value of the ROC curve was 0.903, thus displaying a high accuracy and sensitivity for the model (**Figure 4A**). The Kaplan–Meier curve showed a significantly improved overall survival in the low-risk group (**Figure 4B**, left; *p* < 0.001), a finding consistent with the validation cohort GSE31684 (**Figure 4B**, right; *p* = 0.011). Subsequently, univariate and multivariate Cox proportional risk analyses were performed on the TCGA cohort. The results positioned the risk score of the prognosis model as an independent prognostic factor, with a high-risk score associated with a poor prognosis (**Figure 4C**). The validation set produced analogous results (**Figure 4D**).

We further sought to uncover any potential ties between the risk score and the immune landscape. With the CIBERSORT algorithm we estimated the relative proportions of 22 distinct immune cells for each patient in the high- and low-risk groups in the TCGA dataset. The radar plots illustrate the disparities in various immune cells between the two groups (**Figure S3A**). We found that the infiltration levels of M0 macrophages, M2 macrophages, neutrophils, activated mast cells, and resting memory CD4 T cells were higher in the high-risk group (*p* < 0.05), while the infiltration levels of regulatory T cells (Treg), CD8 T cells, T follicular helper cells, plasma cells, and activated memory CD4 T cells were lower in the high-risk group (**Figure S3B**; *p* < 0.05).

Overall, these findings endorsed the reliability and sensitivity of our constructed risk model, firmly positioning the risk score as an independent prognostic factor for BLCA patients. Meanwhile, the results highlighted differences in the infiltration levels for several immune cells between the risk groups.

### 3.5 Common DEG analysis across differential TMB/IRGPs-derived risk groups

**Figure 5:**
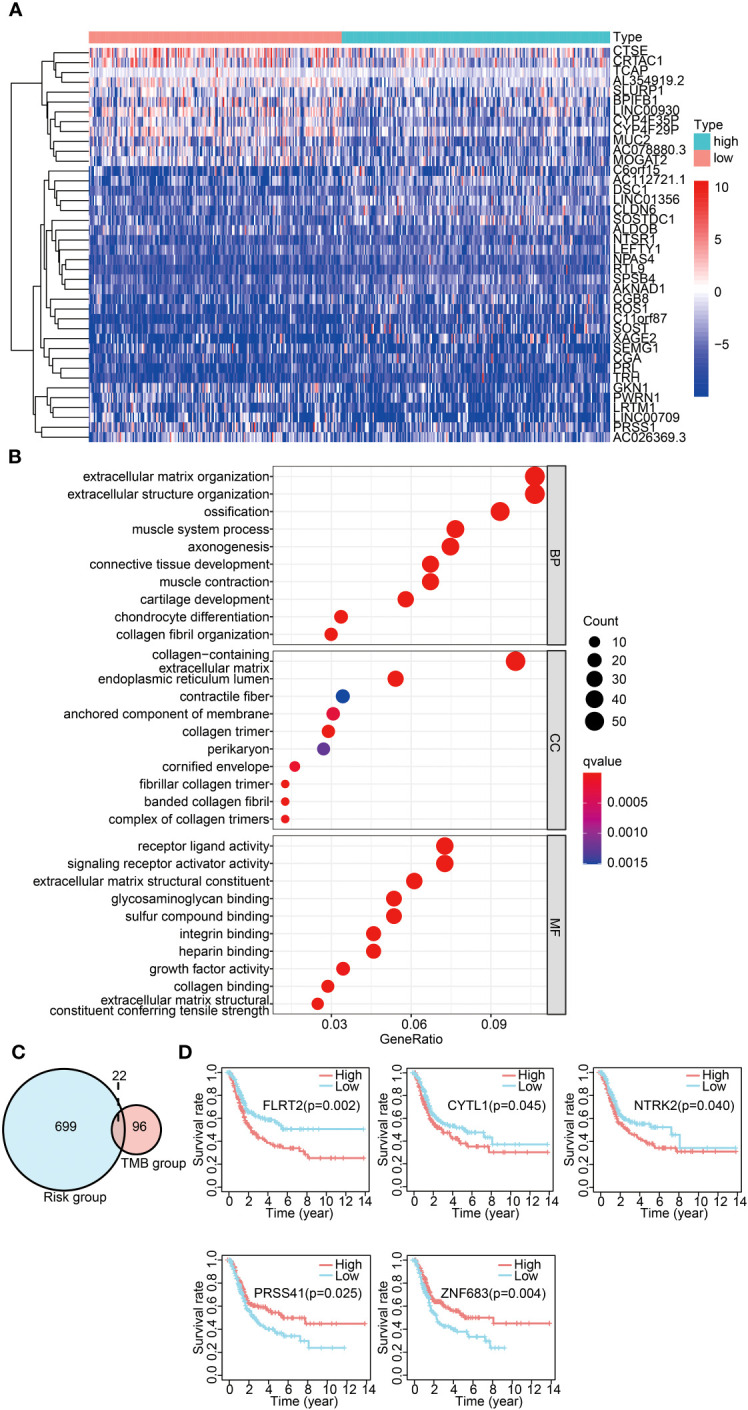
Common DEG analysis across differential TMB/IRGPs-derived risk groups. **(A)** Heatmap displaying the top 40 DEGs between the high- and low-risk groups. **(B)** GO analysis of DEGs in three categories (BPs, MFs, and CCs). BPs, biological processes; MFs, molecular functions; CCs, cellular components. **(C)** Venn diagram of common DEGs between different TMB groups and different IRGP-derived risk groups. **(D)** Survival analysis of five significantly related genes with Kaplan–Meier survival curves.

To identify the critical immune-related genes that may influence prognosis, we investigated the DEGs in the high- and low-risk groups. The DEG landscape was illustrated in a volcano plot (**Figure S2B**), while the top 40 DEGs were comprehensively portrayed in a heatmap (**Figure 5A**). The GO terms included three distinct domains: biological processes (BPs), molecular functions (MFs), and cellular components (CCs). The first 10 enrichment terms across each category are displayed in **Figure 5B**. We found that these DEGs were involved in the BPs such as extracellular matrix and structure formation, collagen production, and receptor ligand activity.

A significant difference was observed in the prognosis analysis between both high- and low-TMB groups and the high- and low-risk groups. To delve into the relationship between these groups, we investigated common DEGs across the high- and low-TMB groups and the high- and low-risk groups. The Venn diagram displaying the overlap of the DEGs is presented in **Figure 5C**. A total of 22 common DEGs were identified from this analysis. The results indicated that *FLRT2*, *NTRK2*, *CYTL1*, *ZNF683*, and *PRSS41* genes were significantly associated with prognosis using univariate Cox analysis (**Table 1**). Furthermore, we formulated a multivariate Cox model and conducted a Kaplan–Meier survival curve analysis, which confirmed the significant associations of *FLRT2* (*p* = 0.002), *NTRK2* (*p* = 0.04), *CYTL1* (*p* = 0.045), *ZNF683* (*p* = 0.004), and *PRSS41* (*p* = 0.025) genes with overall survival in BLCA patients (**Figure 5D**). Notably, patients exhibiting low expression levels of the *FLRT2*, *NTRK2*, and *CYTL1* genes had improved survival rates, while higher expression levels of *ZNF683* and *PRSS41* were associated with superior survival outcomes. In conclusion, our integrative analysis of TMB and IRGPs led to the identification of five key genes with significant prognostic relevance.

**Table 1:** *FLRT2*, *NTRK2*, *CYTL1*, *ZNF683*, and *PRSS41* genes were significantly associated with prognosis as determined by univariate Cox analysis.

Gene	HR	HR.95L	HR.95H	*p*-value
*NTRK2*	1.0509	1.0167	1.0862	0.0032
*FLRT2*	0.7742	0.6152	0.9741	0.0290
*ZNF683*	1.0136	1.0026	1.0247	0.0146
*CYTL1*	0.9227	0.8517	0.9997	0.0491
*PRSS41*	1.3954	1.2026	1.6190	1.1156e-05

### 3.6 Prognostic significance and functional analysis of *FLRT2*

**Figure 6:**
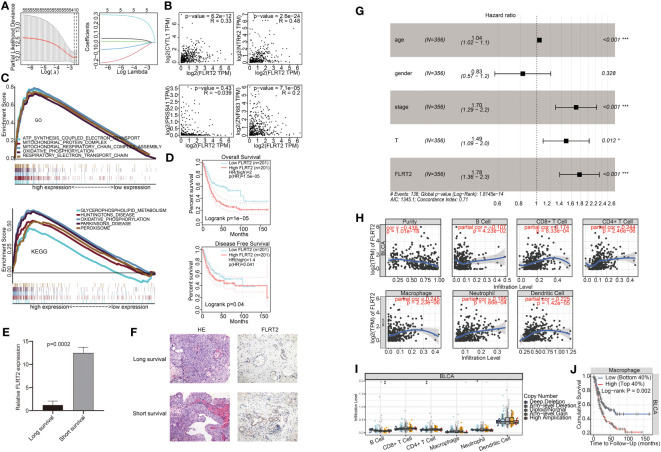
Correlation of *FLRT2* expression with BLCA prognosis and immune cell infiltration. **(A)** Lasso regression analysis of *FLRT2*, *NTRK2*, *CYTL1*, *ZNF683*, and *PRSS41*. Left panel: 10-fold cross-validation result identifying optimal values of the penalty parameter λ; right panel: lasso coefficient profiles of the five significantly related genes. **(B)** Spearman correlation analysis between the expression level of *FLRT2* and *NTRK2*, *CYTL1*, *ZNF683*, and *PRSS41* using GEPIA. **(C)** Top 5 significantly different pathways of the GO (top panel) and KEGG (bottom panel) enrichment analysis as displayed in GSEA enrichment score plots. GSEA, gene set enrichment analysis; GO, Gene Ontology; KEGG, Kyoto Encyclopedia of Genes and Genomes. **(D)** Survival analysis of *FLRT2* expression with OS and DFS using GEPIA. Top panel: correlation of *FLRT2* expression with OS; bottom panel: correlation of *FLRT2* expression with DFS. **(E)** The mRNA expression levels of *FLRT2* gene in the long-survival and short-survival patient group as determined by RT-PCR. Long survival, overall survival > 5 years; short survival, overall survival < 2 years. **(F)** Representative HE (200×) and IHC (200×) images of tumor samples from the long-survival and short-survival patient group. Long survival, overall survival > 5 years; short survival, overall survival < 2 years. **(G)** Multivariate Cox regression analysis of the prognostic factors in the TCGA cohort. ***p < 0.001; **p < 0.01; *p* < 0.05. **(H)** TIMER Spearman correlation analysis between *FLRT2* expression and immune cell infiltration levels. **(I)** Differences in infiltration levels of immune cells with different CNVs of the *FLRT2* gene using TIMER. ***p < 0.001; **p < 0.01; *p < 0.05; •*p* < 0.1. **(J)** Kaplan–Meier plots displaying the impact of macrophage infiltration levels on BLCA prognosis.

To identify the key genes among *FLRT2*, *NTRK2*, *CYTL1*, *ZNF683*, and *PRSS41*, we performed lasso regression analysis. Each gene was identified as an independent variable with a coefficient trajectory (**Figure 6A**, left). We then performed 10-fold cross-validation to assess the accuracy of this risk model and obtained confidence intervals under each log(λ) value (**Figure 6A**, right). This analysis brought the key gene *FLRT2* into focus. We conducted a Spearman correlation analysis based on GEPIA, examining the correlation of *FLRT2* with the other genes. The results revealed that *FLRT2* had a significant positive correlation with *NTRK2*, *CYTL1*, and *ZNF683* genes, while no association with *PRSS41* was found (**Figure 6B**). *PRSS41* expression was observed to significantly correlate with *ZNF683* expression (**Figure S4**). GSEA enrichment analysis was then performed to explore significantly enriched signaling pathways related to *FLRT2*. The top five significantly enriched signaling pathways are presented in **Figure 6C**. The GSEA enrichment scores of GO and KEGG analysis manifested that *FLRT2* was important with mitochondrial and peroxisome function in BLCA patients.

Subsequently, a comprehensive analysis was undertaken on the *FLRT2* gene. BLCA patients were categorized into high- and low-expression groups based on *FLRT2* expression levels. Survival analysis about OS and disease-free survival (DFS) rates were performed using GEPIA. The patients in the low *FLRT2* expression group presented an improved OS rate (**Figure 6D**, top; *p* = 0.04) and DFS rate (**Figure 6D**, bottom; *p* = 1e-05). To substantiate the prognostic significance of *FLRT2* in BLCA patients, we assessed its expression levels in tumor samples from six BLCA patients with varying survival durations through RT-PCR and immunohistochemical (IHC) analysis. Our analysis revealed that the mRNA expression levels of *FLRT2* were significantly higher in the short-survival patient group (overall survival < 2 years) than in those in long-survival patient group (overall survival > 5 years) (*p* = 0.0002; **Figure 6E**). Similarly, IHC analysis of paraffin-embedded tumor samples from the short-survival patient group demonstrated increased levels of *FLRT2* gene expression (**Figure 6F**), corroborating the RT-PCR results. These observations collectively indicated that elevated expression of *FLRT2* was associated with a poorer survival prognosis in BLCA patients. We then performed a multivariate Cox regression analysis and the results identified the *FLRT2* gene as a high-risk factor and an independent prognostic marker for BLCA patients [hazard ratio (HR) 1.78, 95% CI 1.36 to 2.3; *p* < 0.001] (**Figure 6G**).

Furthermore, to ascertain whether gene expression and CNVs of the *FLRT2* gene influence immune cell infiltration, we investigated the relationship between *FLRT2* and immune cell infiltration. Using a Spearman analysis of TIMER, we found that *FLRT2* expression was statistically significant correlated with tumor purity, B cells, CD8^+^ T cells, CD4^+^ T cells, macrophages, neutrophils, and dendritic cells (**Figure 6H**; *p* < 0.05). Further analysis disclosed that CNV amplification of the *FLRT2* gene was significantly associated with B-cell infiltration and CNV deletion of *FLRT2* was significantly correlated with CD4^+^ T cell and neutrophil infiltration (**Figure 6I**; *p* < 0.05). The relationship between CNV levels of *NTRK2*, *CYTL1*, *ZNF683*, and *PRSS41* and immune cell infiltration are depicted in **Figure S5**. We also explored the impact of immune cells on overall survival, and the results are displayed in **Figures 6J**, **S6**. Notably, only macrophage infiltration level was found to be significantly associated with overall survival in BLCA patients (*p* = 0.002), with high levels of macrophage infiltration correlating with a poor prognosis.

In summary, the results suggested that *FLRT2* was a high-risk and an independent prognostic factor in BLCA patients. *FLRT2* expression appeared to modulate the expression of other genes, including *NTRK2*, *CYTL1*, *ZNF683*, and *PRSS41* either directly or indirectly. Additionally, we found that *FLRT2* gene expression may affect the overall survival of BLCA patients by regulating the levels of macrophage infiltration.

### 3.7 The methylation landscape of *FLRT2*

**Figure 7:**
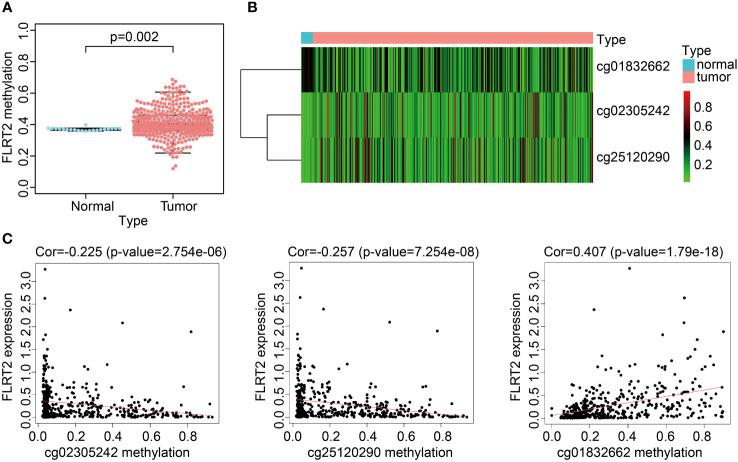
*FLRT2* methylation landscape. **(A)** Scatterplot showing *FLRT2* differential methylation between the normal and tumor groups. **(B)** Heatmap exhibiting site-specific methylation differences in the tumor and normal groups. **(C)** Correlation analysis between site-specific methylation and *FLRT2* gene expression.

To delve into the potential mechanisms impacting *FLRT2* expression, we considered the role of methylation, as it had been reported to modulate *FLRT2* expression. We analyzed the methylation levels in the TCGA-BLCA cohort, finding a slight increase in methylation in BLCA patients compared with the control cohort (0.41 vs 0.37; *p* = 0.002) (**Figure 7A**). Subsequently, we explored the correlation between *FLRT2* methylation and expression level, but no statistically significant relationship was uncovered (**Figure S7**). We further examined the site-specific methylation of *FLRT2* in the tumor cohort. Compared with the control cohort, the sites of cg25120290 and cg02305242 displayed hypermethylation, whereas the site cg01832662 was hypomethylated in the tumor group (**Figure 7B**). An analysis of the relationship between the differential methylation status at sites cg25120290, cg02305242, and cg01832662 and the *FLRT2* gene expression revealed that methylation at sites cg25120290 (cor = −0.257; *p* = 7.254e-08) and cg02305242 (cor = −0.225; *p* = 2.754e-06) was negatively correlated with *FLRT2* expression. Conversely, methylation at site cg01832662 was positively correlated with *FLRT2* expression (cor = 0.407; *p* = 1.79e-18) (**Figure 7C**). In conclusion, the combination of hypermethylation at sites cg25120290 and cg02305242 and hypomethylation at site cg01832662 resulted in diminished *FLRT2* gene expression, resulting in a favorable prognosis for BLCA patients.

## 4 Discussion

Bladder cancer (BLCA) is a prevalent malignant tumor in the urinary system (1). The mortality and incidence rates of bladder cancer are increasing in countries, such as the United States and China (2, 3). Despite advancements in immunotherapy, the prognosis for BLCA remains poor, and recurrence is common (23–27). Therefore, identification of relevant prognostic predictors is crucial for improving disease management, treatment approaches, and prognosis. In this study, we constructed a prognostic risk model with high accuracy using IRGPs. We then analyzed TMB and IRGPs, and identified a key prognosis gene, *FLRT2*, in BLCA patients.

Previous studies have shown that TMB is an effective biomarker for predicting responses to immunotherapy, with a higher TMB correlating with improved outcomes in BLCA patients (28, 29). Consistent with these findings, we also observed that patients with a high TMB had an improved overall survival. However, no association between TMB-derived immune-related DEGs and BLCA prognosis was found in this study. Therefore, we analyzed the IRGPs in bladder cancer patients. The constructed prognostic risk model using IRGPs exhibited an AUC value of 0.903, thus indicating high accuracy and sensitivity. The model-derived risk score was further verified as an independent prognostic factor for BLCA patients using multivariate Cox regression analysis. Additionally, the validation cohort (GSE31684) was analyzed and the results were consistent with this finding. Thus, this study succeeded in constructing a highly accurate prognostic model for BLCA patients, and the risk score derived from this model offered a reliable approach to predicting BLCA patient prognosis. A previous study identified two 5-methylcytosine (5mC) clusters, including 5mC cluster 1 and cluster 2, in BLCA (30). This finding provided an avenue for constructing robust models using 5mc subtypes. On this basis, we will evaluate the effectiveness of our model for different molecular types of BLCA, and construct innovative models in future research.

According to this risk prediction model, BLCA patients were categorized into high- and low-risk groups, and we investigated the DEGs between these two groups in combination with the DEGs in the the high- and low-TMB groups. Through Cox regression analysis we identified five genes associated with prognosis, including *FLRT2*, *NTRK2*, *CYTL1*, *ZNF683*, and *PRSS41*. Among them, *NTRK2* was identified as an oncogene in 1982 by Mariano Barbacid and colleagues (31). *CYTL1* mediates proangiogenic functions attributed to endothelial progenitor cells (such as ECFC) *in vivo* and may be a candidate to support angiogenesis and tissue regeneration in ischemic pathology (32). *ZNF683* is a transcription factor that mediates the transcriptional program in various innate and adaptive immune tissue-resident lymphocyte T-cell types, such as tissue-resident memory T (Trm), natural killer (trNK), and natural killer T (NKT) cells (33, 34). In addition, *PRSS41* is a kind of serine protease. *FLRT2* has been shown to participate in cell–cell adhesion, cell migration, and axon guidance. These five genes have important functions in the process of cancer development, angiogenesis, and immune cell regulation. Using lasso regression analysis, *FLRT2* was identified as the most crucial gene. Its expression was found to be significantly correlated with the other four genes directly or indirectly.

Cai et al. (35) and Hu et al. (36) revealed the critical significance of BCAT2 and Siglec15 in the tumor microenvironment of BLCA patients. In this study, we identified a novel prognostic gene and clarified its functional mechanisms in BLCA patients. A significant association was observed between lower levels of *FLRT2* expression and improved survival outcomes in BLCA patients. Through multivariable Cox analysis, *FLRT2* emerged as an independent prognostic factor for BLCA patients. Furthermore, a positive correlation was found between *FLRT2* expression and macrophage cell infiltration. Additionally, lower levels of macrophage infiltration correlated with improved BLCA patient survival. These findings revealed that *FLRT2* had a potential impact on BLCA prognosis by modulating macrophage cell infiltration. Notably, hypermethylation at sites cg25120290 and cg02305242, combined with hypomethylation at site cg01832662 were associated with reduced levels of expression of *FLRT2*. To conclude, we suggested that methylation at these sites led to reduced *FLRT2* expression, influencing macrophage infiltration levels, and, ultimately, improving survival outcomes in BLCA patients.

However, this study has certain limitations. The functional analysis of *FLRT2* was confined to the TCGA-BLCA cohort, without further external validation. To address this limitation, the implementation of molecular biological experiments should be performed for confirming the prognostic significance and function mechanism of the *FLRT2* gene in BLCA patients.

## 5 Conclusions

In this study, we identified *FLRT2* as a novel predictor indicative of poor prognosis in BLCA patients through a comprehensive analysis of TMB and IRGPs. We also revealed that *FLRT2* might influence patient prognosis by modulating macrophage cell infiltration. Notably, hypermethylation at sites cg25120290 and cg02305242, combined with hypomethylation at site cg01832662 correlated with decreased levels of *FLRT2* expression. We inferred that these methylation patterns led to decreased levels of *FLRT2* expression, which potentially contributed to a reduced level of macrophage infiltration, thereby prolonging survival rates in BLCA patients. The identification of *FLRT2* as a predictive biomarker for poor prognosis provides a promising avenue for refining clinical prognosis management and tailoring therapeutic strategies for BLCA patients.

## Data availability statement

The original contributions presented in the study are included in the article/**Supplementary Material**. Further inquiries can be directed to the corresponding author.

## Ethics statement

The studies involving humans were approved by Ethics Committee of Ningbo No.2 Hospital. The studies were conducted in accordance with the local legislation and institutional requirements. The participants provided their written informed consent to participate in this study.

## Author contributions

Conceptualization, YT; investigation and formal analysis, YT and XY; data curation and validation, HC and JL; methodology, YT and QC; visualization, YT and JZ; resources, TC and HD; supervision, TC and QC; funding acquisition, YT, JL, QC and TC; writing – original draft, YT; writing – review & editing, YT, TC and QC.
