# Supplementary material for: Identification of FLRT2 as a key prognostic gene through a comprehensive analysis of TMB and IRGPs in BLCA patients
Source: Front Oncol. 2024 Feb 29;13:1229227. doi: 10.3389/fonc.2023.1229227 (PMC10937436; doi:10.3389/fonc.2023.1229227)
Supplement: Supplementary file 1 [file DataSheet_1.docx]

Supplementary Material

Identification of FLRT2 as a key prognostic gene through a comprehensive analysis of TMB and IRGPs in BLCA patients

Yaling Tao ^1, 2, *^, Xiaoling Yu ^2^, Huaiwei Cong ^2^, Jinpeng Li ^1, 2^, Junqi Zhu ^3^, Huaxin Ding ^4^, Qian Chen ^2, 3, 5, *^ and Ting Cai ^1, 2, *^

^1^ Research Institute, Ningbo No.2 Hospital, Ningbo, China

^2^ Ningbo Institute of Life and Health Industry, University of Chinese Academy of Sciences, Ningbo, China

^3^ Department of Research and Development, Thorgene Co., Ltd., Beijing, China

^4^ Department of Pathology, Ningbo Clinical Pathology Diagnosis Center, Ningbo, China

^5^ Research Institute, Ningbo Hangzhou Bay Hospital, Ningbo, China

*** Correspondence:**

Yaling Tao, taoyaling@ucas.ac.cn

Ting Cai, caiting@ucas.ac.cn

Qian Chen, chenqian@ucas.ac.cn

#
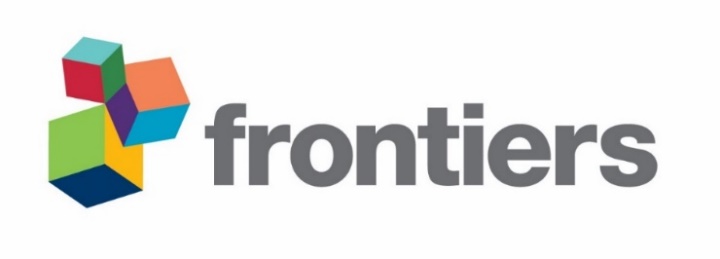


**Supplementary Figures**

**Figure S1** Immune cells infiltration analysis between high- and low- TMB groups. (**A**) Bar plot showing immune infiltration levels in each sample. (**B**) Violin plot presenting levels of immune cells infiltration in high- and low- TMB groups. Green bar: low- TMB group; red bar: high- TMB group; white dot: median value. P values were calculated from the Wilcoxon test.


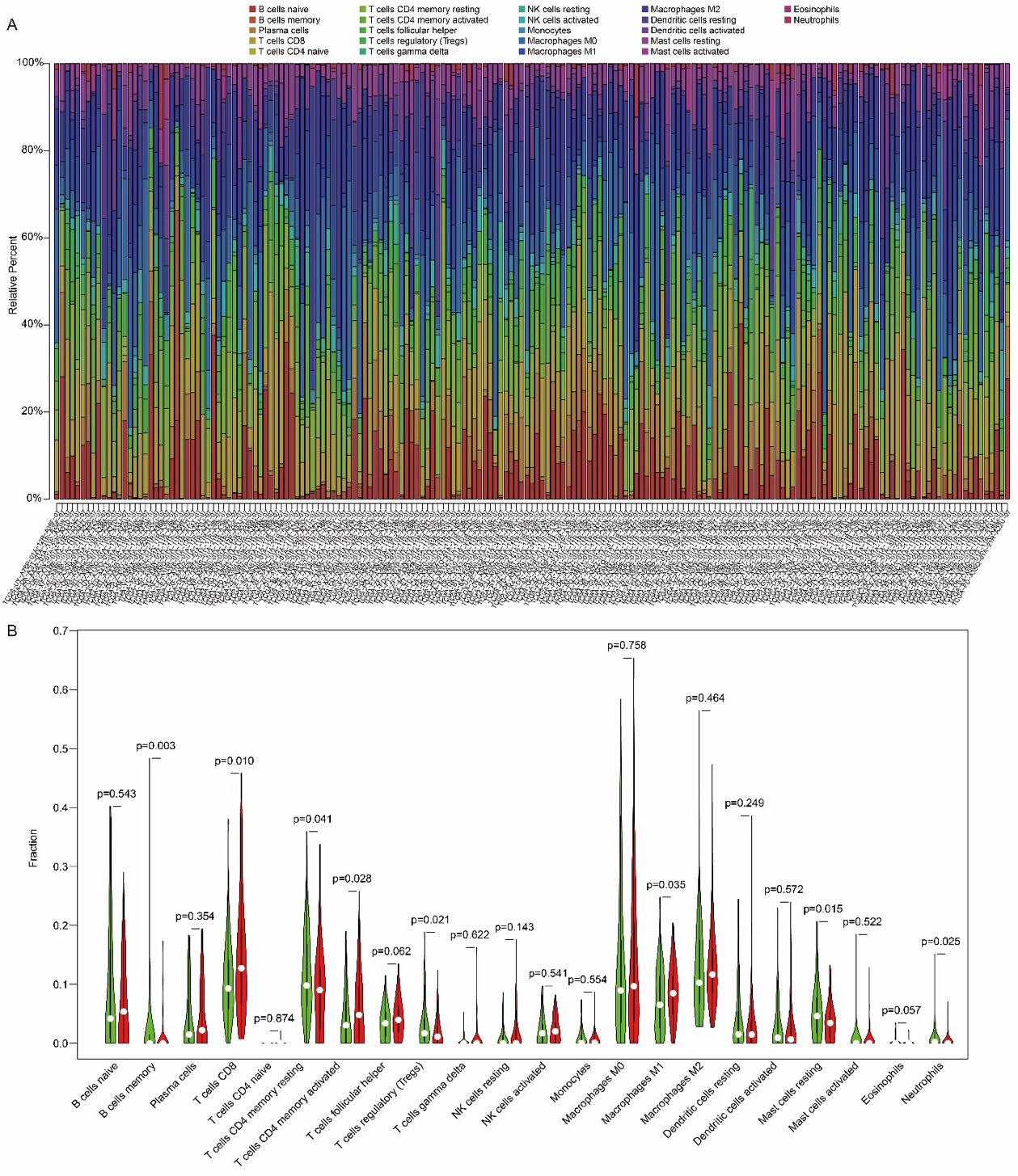


**Figure S2** Volcano plot of DEGs in the high- and low- TMB (**A**) or IRGPs-derived high- and low- risk (**B**) groups.


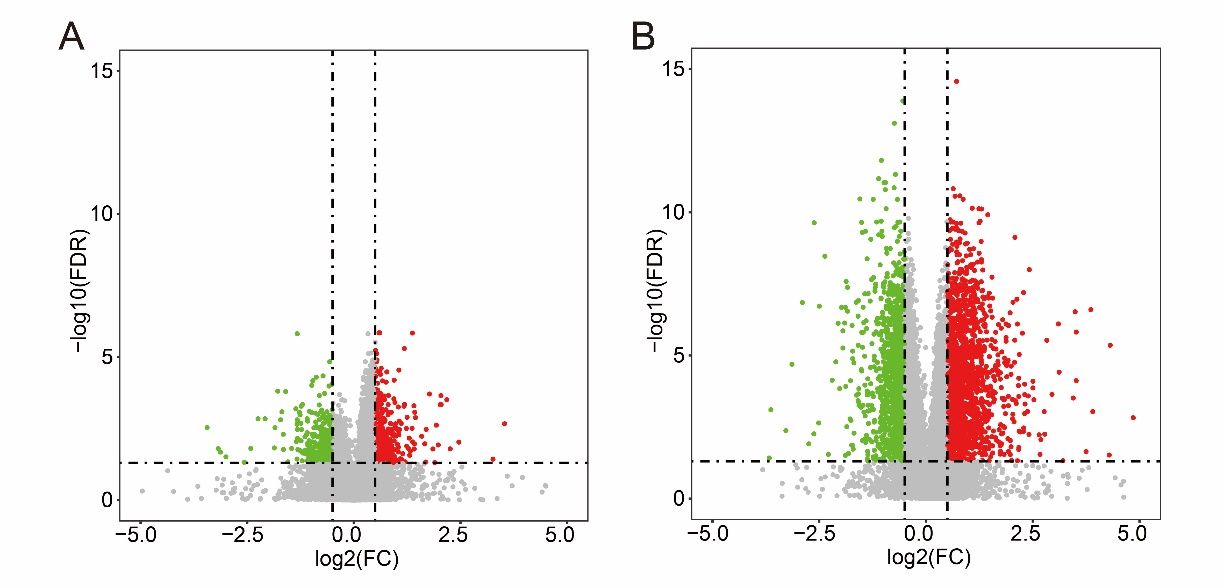


**Figure S3** Immune cells infiltration analysis between high- and low- risk groups. (**A**) The abundance of immune cells estimated by CIBERSORT between the high- and low-risk groups. P-values was based on t-test (*P <0.05, **P < 0.01, ***P < 0.001). (**B**) The bar graph displaying the differential immune cells between the high- and low-risk groups.


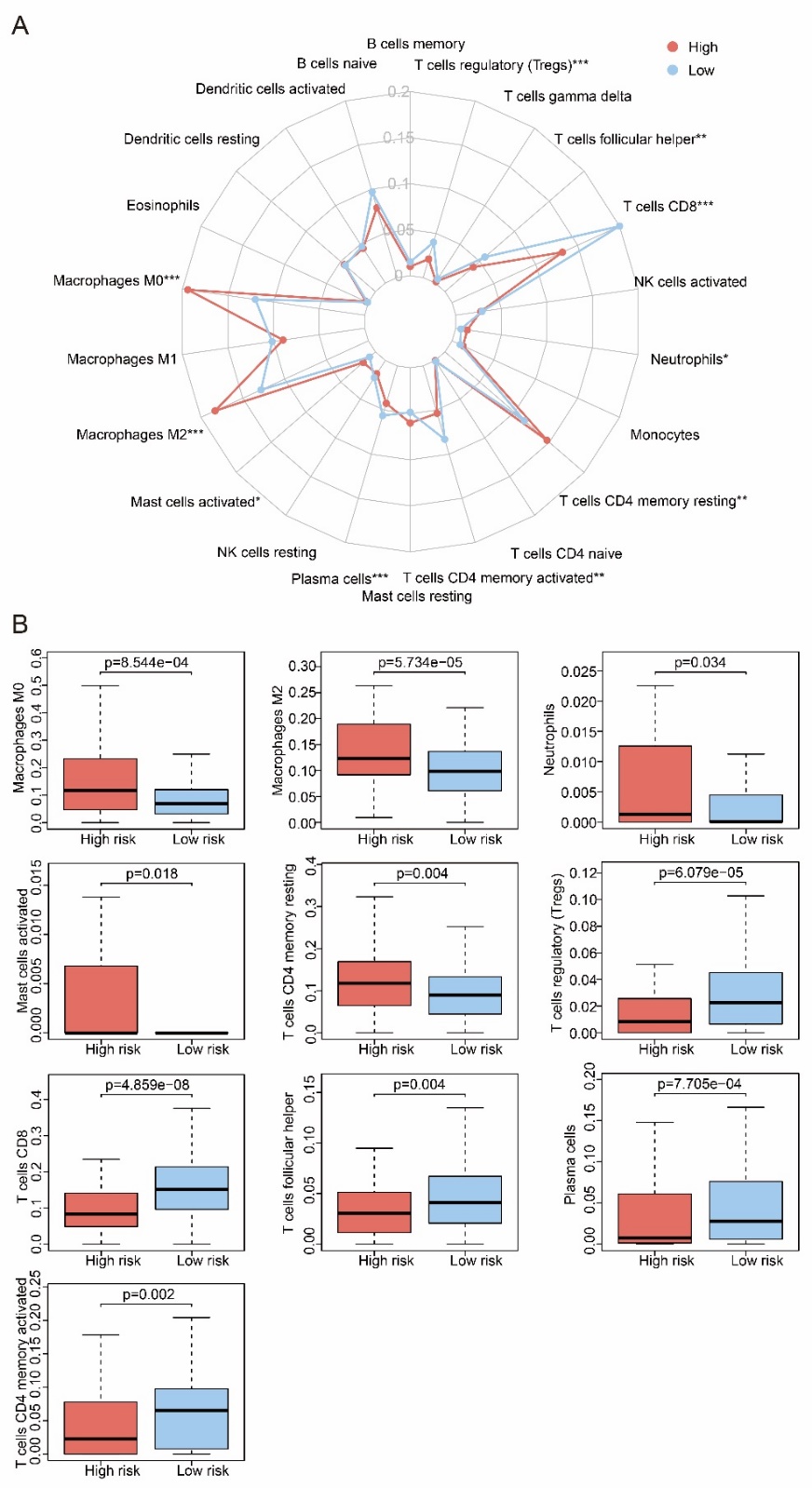


**Figure S4** Correlation analysis of PRSS41 with ZNF683 (**A**), NTRK2 (**B**), CYTL1 (**C**) gene expression.


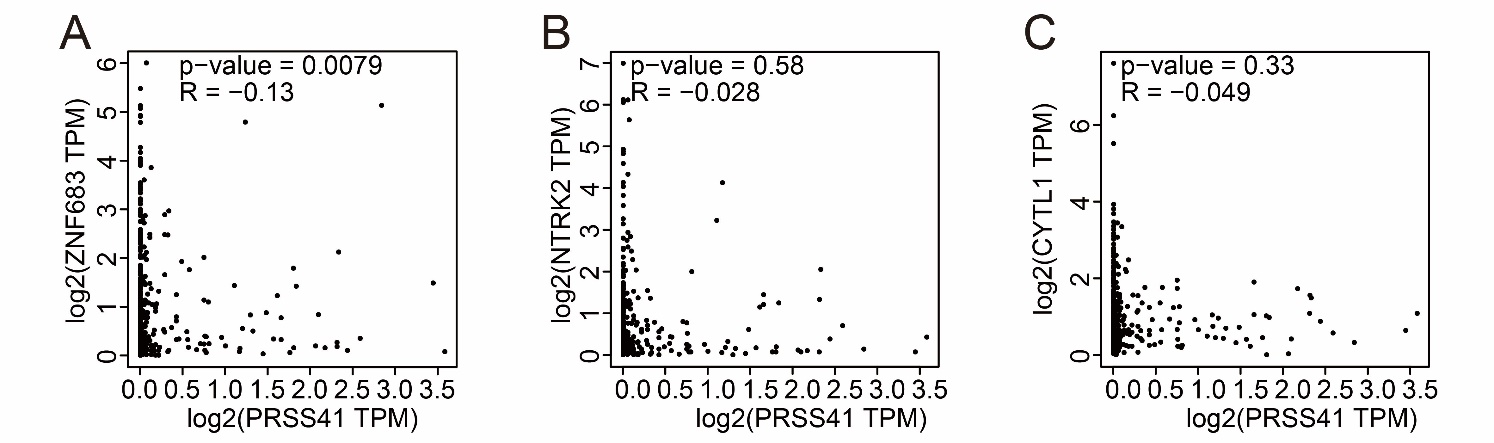


**Figure S5** Immune cell distributions between different CNV types of CYTL1 (**A**), NTRK2 (**B**), PRSS41 (**C**), ZNF683 (**D**).


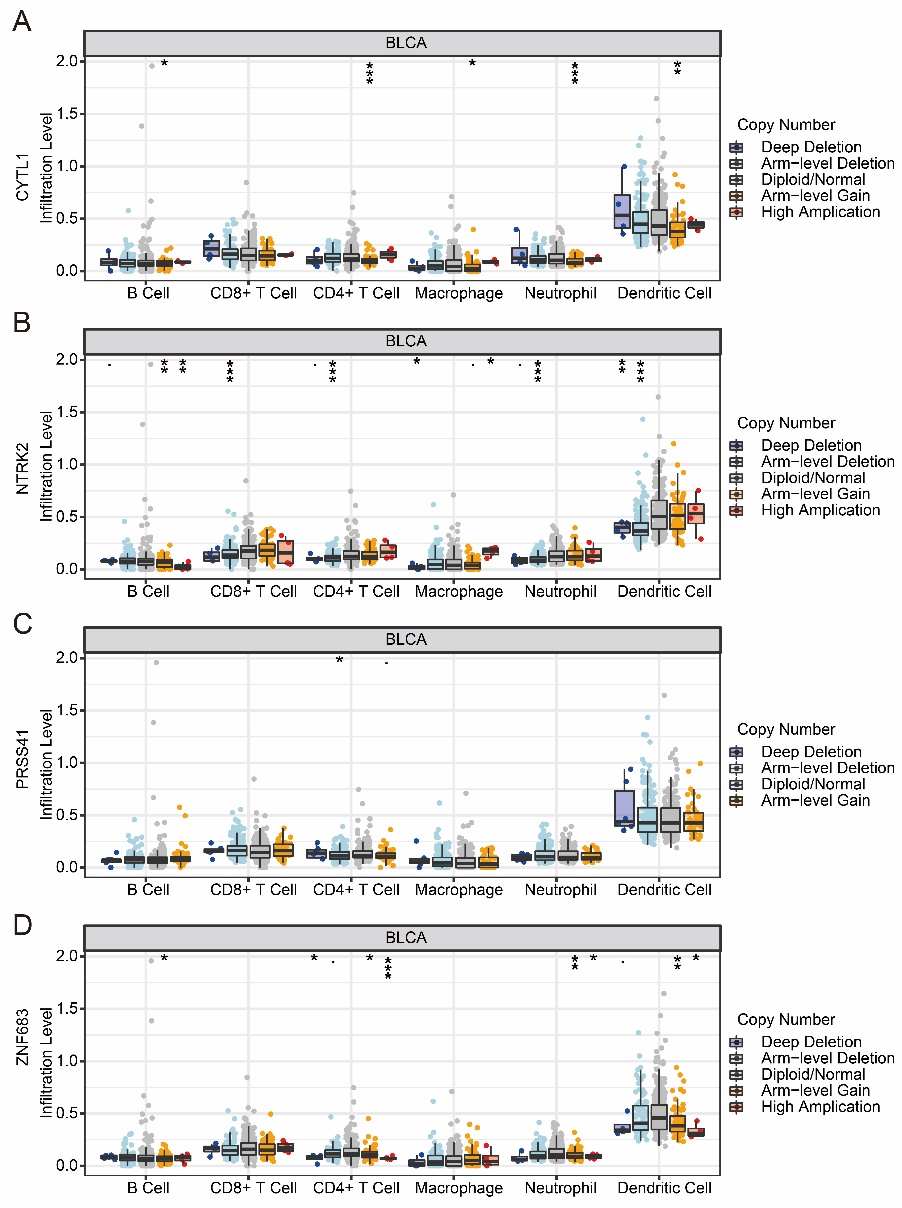


**Figure S6** Prognostic correlation analysis of immune cells.


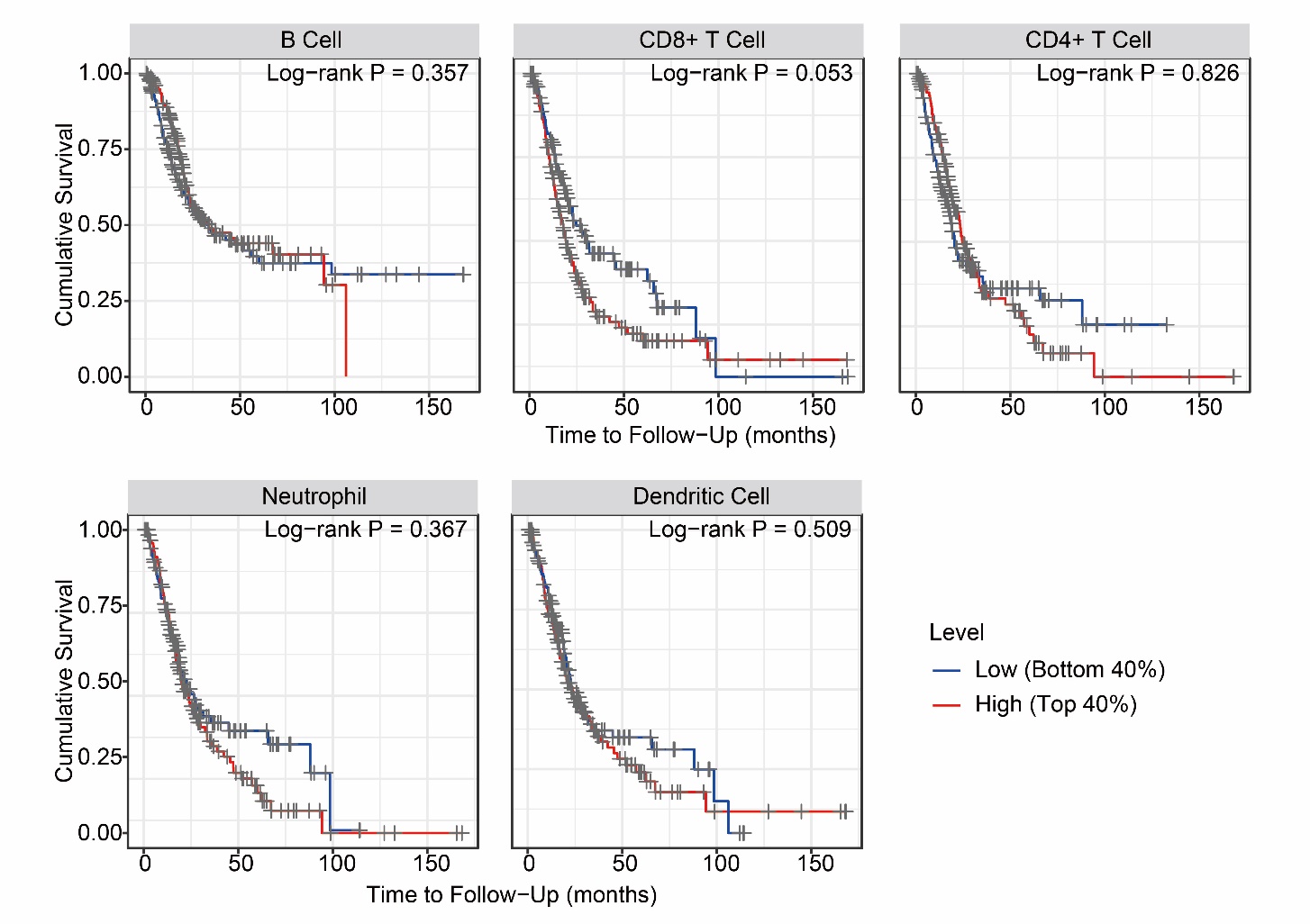


**Figure S7** Correlation analysis between FLRT2 methylation and expression level.


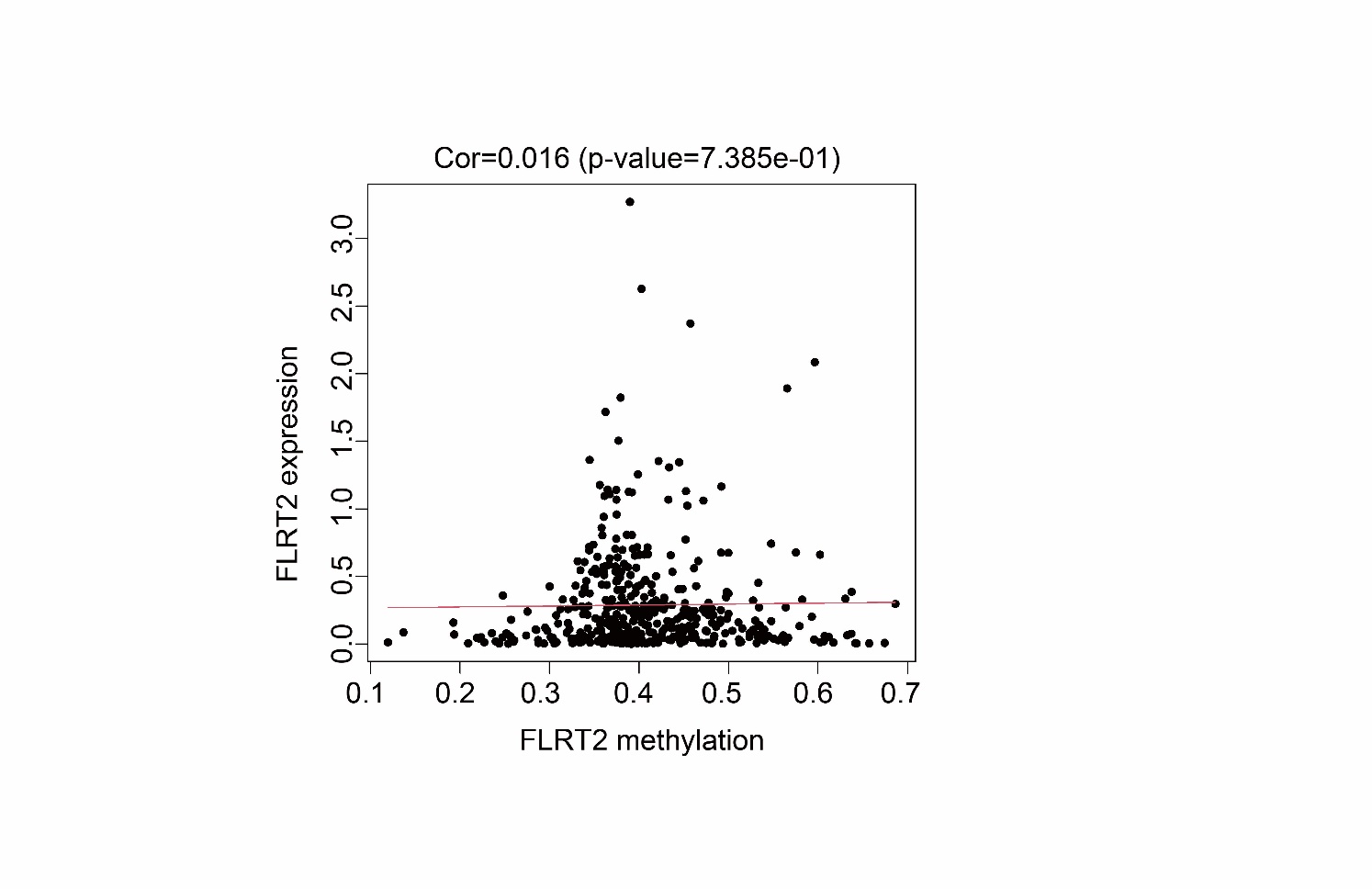


## Supplementary Tables

**Table S1** Immune gene pairs.

| IRG1 | Full name | Immune processes | IRG2 | Full name | Immune processes | Coefficient |
| --- | --- | --- | --- | --- | --- | --- |
| CTSE | Cathepsin E | Antigen_Processing_and_Presentation | CKLF | Chemokine like factor | Chemokines | -0.03954 |
| CTSE | Cathepsin E | Antigen_Processing_and_Presentation | PTN | pleiotrophin | Cytokines | -0.00037 |
| CTSE | Cathepsin E | Antigen_Processing_and_Presentation | TNFRSF14 | TNF receptor superfamily member 14 | Cytokine_Receptors | -0.08856 |
| MICA | MHC class I polypeptide -related sequence A | Natural Killer_Cell_Cytotoxicity | LTBP2 | Latent transforming growth factor beta binding protein 2 | Cytokines | -0.16844 |
| PSMD10 | Proteasome 26S subunit, non-ATPase 10 | Antigen_Processing_and_Presentation | OAS1 | 2'-5'-oligoadenylate synthetase 1 | Antimicrobials | 0.020358 |
| PSMD11 | Proteasome 26S subunit, non-ATPase 11 | Antigen_Processing_and_Presentation | OAS1 | 2'-5'-oligoadenylate synthetase 1 | Antimicrobials | 0.040266 |
| RFXANK | Regulatory factor X associated ankyrin containing protein | Antigen_Processing_and_Presentation | IRF3 | Interferon regulatory factor 3 | Antimicrobials | 0.49908 |
| CXCL10 | C-X-C motif chemokine ligand 10 | Antimicrobials | PTHLH | Parathyroid hormone like hormone | Cytokines | -0.02338 |
| CXCL11 | C-X-C motif chemokine ligand 11 | Cytokines | MMP9 | Matrix metallopeptidase 9 | Antimicrobials | -0.22698 |
| CXCL13 | C-X-C motif chemokine ligand 13 | Cytokines | CKLF | Chemokine like factor | Chemokines | -0.20408 |
| CXCL13 | C-X-C motif chemokine ligand 13 | Cytokines | TNFRSF1B | TNF receptor superfamily member 1B | Cytokine_Receptors | -0.10495 |
| CCL13 | C-X-C motif chemokine ligand 13 | Cytokines | PTHLH | Parathyroid hormone like hormone | Cytokines | -0.04388 |
| ZC3HAV1L | Zinc finger CCCH-type containing, antiviral 1 like | Antimicrobials | VAV3 | Vav guanine nucleotide exchange factor 3 | BCR Signaling Pathway | 0.146389 |
| IFNGR1 | Interferon gamma receptor 1 | Cytokine_Receptors | CDK4 | Cyclin dependent kinase 4 | TCR Signaling Pathway | -0.03167 |
| A2M | Alpha-2-macroglobulin | Antimicrobials | PPARG | Peroxisome proliferator activated receptor gamma | Antimicrobials | 0.003784 |
| APOBEC3G | Apolipoprotein B mRNA editing enzyme catalytic subunit 3G | Antimicrobials | CMTM8 | CKLF like MARVEL transmembrane domain containing 8 | Cytokines | -0.05151 |
| TLR2 | Toll like receptor 2 | Antimicrobials | PDK1 | Pyruvate dehydrogenase kinase 1 | TCR Signaling Pathway | -0.41055 |
| IL1B | Interleukin 1 beta | Antimicrobials | PTX3 | Pentraxin 3 | Antimicrobials | -0.16095 |
| DDX58 | DExD/H-box helicase 58 | Antimicrobials | OSMR | Oncostatin M receptor | Cytokine_Receptors | -0.03908 |
| OASL | 2'-5'-oligoadenylate synthetase like | Antimicrobials | TNC | Tenascin C | Cytokines | -0.06909 |
| APOD | Apolipoprotein D | Antimicrobials | IRF9 | Interferon regulatory factor 9 | Antimicrobials | 0.036978 |
| APOD | Apolipoprotein D | Antimicrobials | TNFSF13B | TNF superfamily member 13b | Cytokines | 0.181962 |
| MAPK1 | Mitogen-activated protein kinase 1 | Natural Killer_Cell_Cytotoxicity | EGFR | Epidermal growth factor receptor | Cytokine_Receptors | -0.14613 |
| LRP1 | LDL receptor related protein 1 | Antimicrobials | CD40 | CD40 molecule | Antimicrobials | 0.016666 |
| LRP1 | LDL receptor related protein 1 | Antimicrobials | SDC3 | Syndecan 3 | Cytokine_Receptors | 0.036791 |
| LRP1 | LDL receptor related protein 1 | Antimicrobials | ITGB2 | Integrin subunit beta 2 | Natural Killer_Cell_Cytotoxicity | 0.197809 |
| NEDD4 | NEDD4 E3 ubiquitin protein ligase | Antimicrobials | BLNK | B cell linker | BCR Signaling Pathway | 0.026647 |
| VEGFA | Vascular endothelial growth factor A | Antimicrobials | LYN | LYN proto-oncogene, Src family tyrosine kinase | BCR Signaling Pathway | -0.36112 |
| BPHL | Biphenyl hydrolase like | Antimicrobials | BLNK | B cell linker | BCR Signaling Pathway | 0.096262 |
| TYK2 | Tyrosine kinase 2 | Antimicrobials | LTBP1 | Latent transforming growth factor beta binding protein 1 | Antimicrobials | -0.02747 |
| TYK2 | Tyrosine kinase 2 | Antimicrobials | IGF2R | Insulin like growth factor 2 receptor | Cytokine_Receptors | -0.09563 |
| TCF7L2 | Transcription factor 7 like 2 | Antimicrobials | LTBP2 | Latent transforming growth factor beta binding protein 2 | Cytokines | -0.00572 |
| DCK | Deoxycytidine kinase | Antimicrobials | GMFB | Glia maturation factor beta | Cytokines | -0.19078 |
| DCK | Deoxycytidine kinase | Antimicrobials | LTBP2 | Latent transforming growth factor beta binding protein 2 | Cytokines | -0.01487 |
| CSK | C-terminal Src kinase | Antimicrobials | MAP2K1 | Mitogen-activated protein kinase kinase 1 | Antimicrobials | -0.18357 |
| IL18 | Interleukin 18 | Antimicrobials | EGFR | Epidermal growth factor receptor | Cytokine_Receptors | -0.07903 |
| IFNAR2 | Interferon alpha and beta receptor subunit 2 | Antimicrobials | DKK1 | Dickkopf WNT signaling pathway inhibitor 1 | Cytokines | -0.27513 |
| PLSCR1 | Phospholipid scramblase 1 | Antimicrobials | OAS1 | 2'-5'-oligoadenylate synthetase 1 | Antimicrobials | 0.154027 |
| BIRC5 | Baculoviral IAP repeat containing 5 | Antimicrobials | EGFR | Epidermal growth factor receptor | Cytokine_Receptors | -0.21943 |
| VCAM1 | Vascular cell adhesion molecule 1 | Antimicrobials | BLNK | B cell linker | BCR Signaling Pathway | 0.053136 |
| GBP2 | Guanylate binding protein 2 | Antimicrobials | LTBP1 | Latent transforming growth factor beta binding protein 1 | Antimicrobials | -0.02893 |
| GBP2 | Guanylate binding protein 2 | Antimicrobials | NAMPT | Nicotinamide phosphoribosyltransferase | Cytokines | -0.2506 |
| OAS1 | 2'-5'-oligoadenylate synthetase 1 | Antimicrobials | BID | BH3 interacting domain death agonist | Natural Killer_Cell_Cytotoxicity | -0.06626 |
| BLNK | B cell linker | BCR Signaling Pathway | PGF | Placental growth factor | Cytokines | -0.06376 |
| CARD11 | Caspase recruitment domain family member 11 | TCR Signaling Pathway | TNC | Tenascin C | Cytokines | -0.05162 |
| PIK3CA | Phosphatidylinositol-4,5-bisphosphate 3-kinase catalytic subunit alpha | Natural Killer_Cell_Cytotoxicity | PTGER4 | Prostaglandin E receptor 4 | Cytokine_Receptors | 0.006921 |
| SEMA4D | Semaphorin 4D | Cytokines | KDR | Kinase insert domain receptor | Cytokine_Receptors | -0.00904 |
| EDNRB | Endothelin receptor type B | Chemokine_Receptors | TNFSF15 | TNF superfamily member 15 | Cytokines | 0.200787 |
| FPR1 | Formyl peptide receptor 1 | Chemokine_Receptors | GZMB | granzyme B | Natural Killer_Cell_Cytotoxicity | 0.020304 |
| CMTM7 | CKLF like MARVEL transmembrane domain containing 7 | Cytokines | CMTM8 | CKLF like MARVEL transmembrane domain containing 8 | Cytokines | -0.07986 |
| CMTM7 | CKLF like MARVEL transmembrane domain containing 7 | Cytokines | IGF2R | Insulin like growth factor 2 receptor | Cytokine_Receptors | -0.00587 |
| CMTM8 | CKLF like MARVEL transmembrane domain containing 8 | Cytokines | CD3D | CD3d molecule | TCR Signaling Pathway | 0.092808 |
| JAG2 | Jagged canonical Notch ligand 2 | Cytokines | EGFR | Epidermal growth factor receptor | Cytokine_Receptors | -0.27186 |
| KITLG | KIT ligand | Cytokines | PRF1 | Perforin 1 | Natural Killer_Cell_Cytotoxicity | 0.112807 |
| LTBP2 | Latent transforming growth factor beta binding protein 2 | Cytokines | INSR | Insulin receptor | Cytokine_Receptors | 0.174538 |
| PDGFD | Platelet derived growth factor D | Cytokines | LCK | LCK proto-oncogene, Src family tyrosine kinase | Natural Killer_Cell_Cytotoxicity | 0.113959 |
| TGFB3 | Transforming growth factor beta 3 | Cytokines | GZMB | Granzyme B | Natural Killer_Cell_Cytotoxicity | 0.007154 |
| APLNR | Apelin receptor | Cytokine_Receptors | ICAM2 | Intercellular adhesion molecule 2 | Natural Killer_Cell_Cytotoxicity | 0.268754 |
| EGFR | Epidermal growth factor receptor | Cytokine_Receptors | THRB | Thyroid hormone receptor beta | Cytokine_Receptors | 0.006872 |
| EGFR | Epidermal growth factor receptor | Cytokine_Receptors | PAK1 | p21 (RAC1) activated kinase 1 | Natural Killer_Cell_Cytotoxicity | 0.021686 |
| KDR | Kinase insert domain receptor | Cytokine_Receptors | PTGER4 | Prostaglandin E receptor 4 | Cytokine_Receptors | 0.081102 |
| KDR | Kinase insert domain receptor | Cytokine_Receptors | MAP3K8 | Mitogen-activated protein kinase kinase kinase 8 | TCR Signaling Pathway | 0.057975 |

**Table S2** Univariate Cox analysis of the genes of GLP1R, KIR2DL4, SSTR5.

| **Gene** | **HR** | **HR.95L** | **HR.95H** | **P value** |
| --- | --- | --- | --- | --- |
| GLP1R | 0.3933 | 0.15898 | 0.9732 | 0.0435 |
| KIR2DL4 | 0.6515 | 0.4784 | 0.8874 | 0.0065 |
| SSTR5 | 0.5490 | 0.3249 | 0.9276 | 0.0250 |
